# Identification of six novel variants in Waardenburg syndrome type II by next‐generation sequencing

**DOI:** 10.1002/mgg3.1128

**Published:** 2020-01-20

**Authors:** Shumin Ren, Xiaojie Chen, Xiangdong Kong, Yibing Chen, Qinghua Wu, Zhihui Jiao, Huirong Shi

**Affiliations:** ^1^ Department of Genetic and Prenatal Diagnosis Center The First Affiliated Hospital of Zhengzhou University Zhengzhou China; ^2^ Department of Cardiovascular Medicine The First Affiliated Hospital of Zhengzhou University Zhengzhou China; ^3^ Department of obstetrics and Gynecology The First Affiliated Hospital of Zhengzhou University Zhengzhou China

**Keywords:** *MITF*, next‐generation sequencing, *SOX10*, Waardenburg syndrome

## Abstract

**Background:**

Waardenburg syndrome (WS) is a dominantly inherited, genetically heterogeneous auditory‐pigmentary syndrome characterized by nonprogressive sensorineural hearing loss and iris discoloration. This study aimed to investigate the underlying molecular pathology in Chinese WS families.

**Methods:**

A total of 13 patients with Waardenburg syndrome type II (WS2) from six unrelated Chinese families were enrolled. We investigated the mutation profile of genes related to congenital deafness in these families through a targeted sequencing technology and validated the candidate variants by Sanger sequencing.

**Results:**

We identified six novel variants in microphthalmia‐associated transcription factor (*MITF*) and SRY‐box 10 (*SOX10*), which were predicted to be disease causing by in silico analysis. Our results showed that mutations in *SOX10* and *MITF* are two major causes of deafness associated with WS, and de novo mutations were frequently found in probands with *SOX10* mutations but not in those with *MITF* mutations.

**Conclusion:**

Results showed that targeted next‐generation sequencing (NGS) enabled us to detect disease‐causing mutations with high accuracy, stability, speed and throughput. Our study extends the pathogenic mutation spectrum of *MITF* and *SOX10*.

## Graphical abstract

We identified six novel variants in *MITF* and *SOX10* gene which predicted to be disease causing by in silico analysis. Our results showed that mutations in *SOX10* and *MITF* are two major causes for deafness associated with WS, and de novo mutations were frequently found in probands with *SOX10* mutations (3/4) but not in those with MITF mutations (0/2).

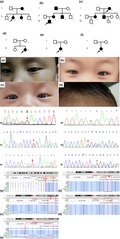

## 1 INTRODUCTION

Waardenburg syndrome (WS) is a clinically and genetically heterogeneous hereditary auditory pigmentary disorder characterized by congenital sensorineural hearing loss and iris discoloration. Many genes have been linked to WS, including paired box 3 (*PAX3*) (OMIM 606597), *MITF* (OMIM 156845), snail family zinc finger 2 (*SNAI2*) (OMIM 602150), endothelin receptor type B (*EDNRB*) (OMIM 131244), endothelin 3 (*EDN3*) (OMIM 131242), and *SOX10* (OMIM 602229). These genes are involved in the formation and development of several types of cells, including pigment‐producing cells called melanocytes. Mutations in any of these genes disrupt the normal development of melanocytes, leading to abnormal pigmentation of the skin (Pingault et al., 2010). Offspring of individuals with WS have a 50% chance of inheriting the pathogenic mutation, and therefore, an exact description of the mutations responsible for the WS is crucial for the genetic counseling of WS patients and their families.

As conventional gene‐by‐gene sequencing is too costly and time‐consuming, recently, next‐generation sequencing (NGS) has been introduced to analyze the exons and flanking intronic regions of genes with clinical relevance. NGS is appropriate for a variety of gene sequencing indications, providing lower turnaround times, lower cost, and a more comprehensive coverage of target regions (Ku et al., 2012; Lee et al., 2014; Lohmann & Klein, 2014; Ng et al., 2010, 2009). We performed NGS to screen all possible genes associated with WS and congenital deafness simultaneously and identified six novel variants in *MITF* and *SOX10* in patients. Further analysis by Sanger sequencing of patients and their parents revealed three de novo occurrence of variants. Our findings show that NGS can be a useful tool for the identification of pathogenic gene variants in WS patients.

## 2 MATERIALS AND METHODS

### 2.1 Ethical compliance

Clinical investigations were conducted according to the Declaration of Helsinki, and the study was approved by the institutional review board of the Medical Ethics Committee of the First Affiliated Hospital of Zhengzhou University.

### 2.2 Patients and samples

**Figure 1:**
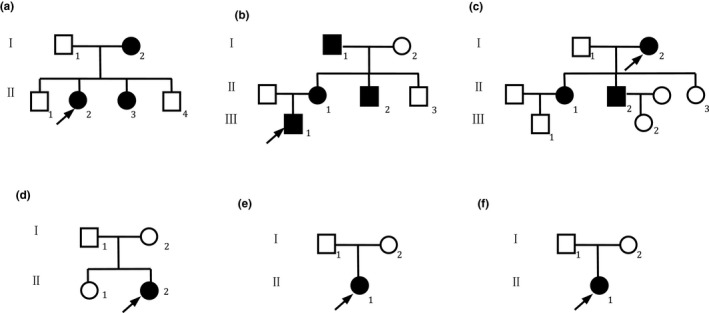
Pedigrees of the Waardenburg syndrome families. Pedigrees of families (a) 01, (b) 02, (c) 03, (d) 04, (e) 05, and (f)06

All patients with WS of Chinese Han nationality were identified at the First Affiliated Hospital of Zhengzhou university (January 2016 to August 2019). Detailed examinations were performed on all patients by medical specialists. The following tests were conducted: observation of skin pigmentation, hair color, joints, skeletomuscular system, digestion, nerves, ophthalmology and otology, and an assessment of intelligence. In addition, a detailed audiological examination was conducted on the probands. The clinical audiology assessment included pure tone test, acoustic immittance, auditory steady‐state response, auditory brainstem response (ABR), otoacoustic emission, test of study ability and psychiatric behavior development, ossa temporale computerized tomography, and magnetic resonance imaging. There were 13 patients with WS (from six families) aged 2 months to 71 years, and nine unaffected family members(WS01‐II:1,WS02‐II:3,WS03‐II:3,WS04‐I:1,WS04‐I:2,WS05‐I:1,WS05‐I:2,WS06‐I:1, and WS06‐I:2) who agreed to take part in the study following audiological and general physical examinations. Among the six families, families WS04 to WS06 were sporadic cases in which the pedigrees did not reveal any history of WS features in the three last generations, while the remaining families had multiple affected individuals (Figure 1). Characteristics of patients are presented in Table 1. Two hundred randomly selected normal individuals were also included in this study. Documented permission of identifiable patient images from patients or legal guardians were obtained by written patient consent. Written informed consent was obtained from all adult subjects and guardians on behalf of the children, prior to clinical evaluation and blood sample collection.

**Table 1:** Summary of clinical data for 13 Chinese WS2 patients

Pedigree	Gender	Age (years)	Hearing loss	Heterochromia of iridum	White forelock	Brown freckles
01‐I:2	Female	60	+	−	−	+
01‐II:2	Female	21	+	−	−	+
01‐II:3	Female	18	+	−	−	+
02‐I:1	Male	57	−	−	−	+
02‐II:1	Female	34	−	−	−	+
02‐II:2	Male	30	−	−	−	+
02‐III:1	Male	7	+	−	−	+
03‐I:1	Female	71	+	+	−	−
03‐II:1	Female	52	+	+(Unilateral)	−	−
03‐II:2	Male	49	+(Unilateral)	+	−	−
04‐II:2	Female	5	+	+	−	−
05‐II:1	Female	5	+	−	+	−
06‐II:1	Female	2/12	+	+	+	−

### 2.3 DNA extraction

Whole genomic DNA was isolated from blood samples using TIANamp Blood DNA Kit (Tiangen Biotech Co., Ltd.) and quantified by an ultraviolet spectrophotometer Du800 (Beckman Coulter, Inc.). The DNA was subsequently stored at −20°C until further use.

### 2.4 Mutational analysis

Genomic DNA was enriched using a customized panel (MyGenostics), which was designed to capture 159 known genes (Table S1) related to hearing loss to detect the genetic cause of the WS families, and then sequenced on the Illumina NextSeq500 system in our clinical laboratory.Raw sequence reads were processed and aligned to the hg19 human reference sequence with the Burrows‐Wheeler Aligner (BWA, version 0.7.5). Single‐nucleotide polymorphism (SNP) and short indel candidates were identified, and these variants were annotated by ANNOVAR to filter SNPs reported in public databases (dbSNP, gnomAD, ESP, Clinvar, 1000 Genomes and ExAC) and the HGMD Professional database. Exonic sequence alterations and intronic variants at exon‐intron boundaries, with unknown frequency or minor allele frequency (MAF) <1% and not present in the homozygous state in those databases were retained. The Sorting Intolerant From Tolerant (SIFT), Polymorphism Phenotyping v2 (PolyPhen‐2, http://genetics.bwh.harvard.edu/pph2/) and MutationTaster (http://www.mutationtaster.org/) algorithms were used to predict the effects of variants on protein function. Nucleotide conservation between species was evaluated using the UCSC Genome Browser Database (https://genome.ucsc.edu/). The candidate variants identified by NGS were confirmed by conventional Sanger sequencing. The carrying situation of the mutations of their family members and 200 randomly selected normal individuals were also tested by Sanger sequencing. Polymerase chain reaction (PCR) primers (Table 2) were designed by Primer premier 5.0 software and were synthesized by Shanghai Shangon Co., Ltd. Capillary electrophoresis apparatus (ABI 3130XL, USA) and the dGTP BigDye® Terminator sequencing kit (ABI, USA) were used for Sanger sequencing.

**Table 2:** Primer pairs of the novel mutations of *MITF* and *SOX10*

Gene exon	Forward primer sequence (5′–3′)	Reverse primer sequence (5′–3′)	Product length
*MITF*‐exon9	GTGCTCTGCCTATTTCAGTGTTTTA	AGGGAGGATTCGCTAACAAGTG	457 bp
*SOX10*‐exon2	GTGGGCGTTGGACTCTTTG	CTACCCTGAATCCACCCGAA	571 bp
*SOX10*‐exon3	CATCTCTCAGTCCACAAATCATAGG	CCATCTCCTGTCTCCACTGACTG	506 bp

## 3 RESULTS

### 3.1 Clinical findings

Before genetic testing, probands WS01 and WS02 were primarily diagnosed with nonsyndromic deafness, and the other four probands (WS03 to 06) were diagnosed with WS2 based on their calculated W index (<1.85) and the absence of musculoskeletal anomalies and intestinal aganglionosis. After genetic diagnosis, further examination of the probands (WS01‐II:2, WS02‐III:1) found scattered freckles on the proband's face, which were previously ignored.

**Figure 2:**
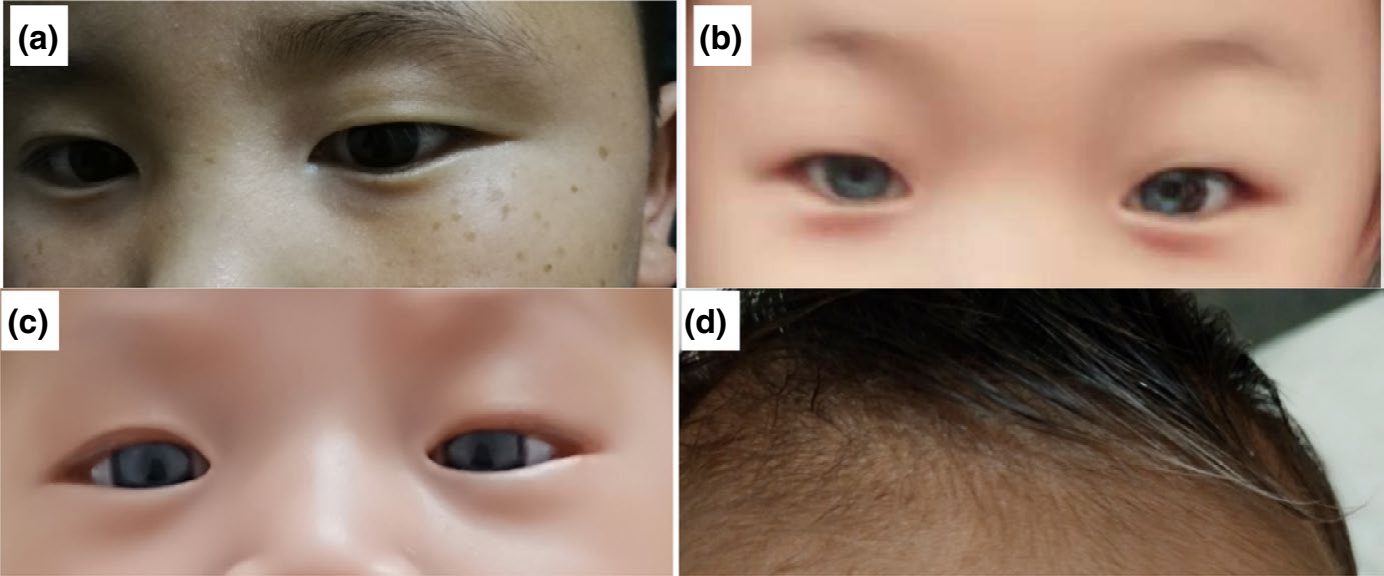
Photographs of partly affected individuals. (a) WS02‐III:1 presented with special brown freckles on the face. (b) WS04‐II:2 presented complete heterochromia iridis. (c, d) WS06‐II:1 presented complete heterochromia iridis and white forelock

Among the 13 WS2 cases, deafness and freckles were the most frequent features. Ten patients (10/13, 76.9%) had sensorineural hearing impairment, seven affected individuals (7/13, 53.8%) had numerous brown freckles on the face, five affected individuals (5/13, 38.5%) had heterochromia iridum and white forelock was observed in two patients (2/13, 15.4%; Figure 2). No patchy or generalized skin depigmentation was observed in any of the patients. Table 1 lists the clinical data of these 13 Chinese WS2 patients.

### 3.2 Identification of mutations and pathogenicity analysis

**Figure 3:**
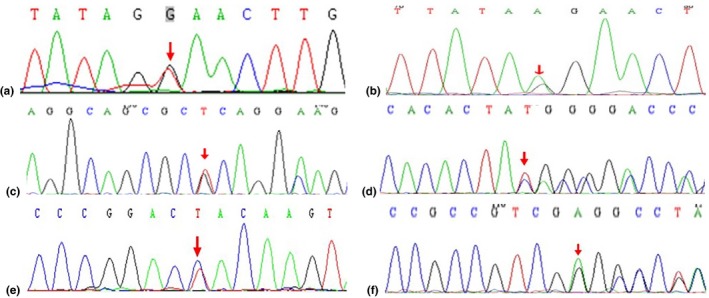
Mutation analyses of Chinese Waardenburg syndrome families 01 to 06. (a) DNA sequence chromatograms presenting heterozygous missense mutation c.859G>T of *MITF* in WS01‐II:2. (b) DNA sequence chromatograms presenting heterozygous missense mutation c.1162‐1G>A of *MITF* in WS02‐III:1. (c) DNA sequence chromatograms presenting heterozygous mutation c.355_356insTCAGGCAGCGC of *SOX10* in WS03‐I:2. (d) DNA sequence chromatograms presenting heterozygous mutation c.1106_1107insTGGGGCCCCCCACACTA of *SOX10* in WS04‐II:2. (e) DNA sequence chromatograms presenting heterozygous mutation c.511T>C of *SOX10* in WS05‐II:1. (f) DNA sequence chromatograms presenting heterozygous mutation c.91_100del of *SOX10* in WS06‐II:1

After screening of all WS‐related genes and congenital deafness, a heterozygous nonsense mutation, c.859G>T, of *MITF* was detected through gene panel sequencing of WS01‐II:2. The variant was predicted to be deleterious by in silico analysis using MutationTaster. We regarded this variant as the best candidate and subsequently validated it by Sanger sequencing. We also tested and verified the mutation in nuclear members of the family. We found that all patients (WS01‐I:2, II:2 and II:3) had the same heterozygous mutation. The family member (WS01‐II:1) with normal phenotype did not have this mutation (Table 3; Figure 3).

**Table 3:** Gene variants of Waardenburg syndrome probands

Pedigree	Gene	Exon	Nucleotide change	Amino acid change	zygosity	Frequency	SITF	PolyPhen2	MutationTaster
01	*MITF*	Exon9	c.859G>T	p.E287X	het	—	—	—	Disease causing
02	*MITF*	intron9	c.859‐1G>A	—	het	—	—	—	Disease causing
03	*SOX10*	Exon2	c.355_356insTCAGGCAGCGC	p.R119Lfs*31	het	—	—	—	Disease causing
04	*SOX10*	Exon4	c.1106_1107insTGGGGCCCCCCACACTA	p.Y369fs	het	—	—	—	Disease causing
05	*SOX10*	Exon3	c.511T>C	p.Y171H	het	—	Damaging	Probably damaging	Disease causing
06	*SOX10*	Exon2	c.91_100del	p.R31Gfs*75	het	—	—	—	Disease causing

* Frequency: from 1000 genomes database. Reference sequence transcript: *MITF*: NM_000248, *SOX10*: NM_006941

A heterozygous splicing mutation, c.859‐1G>A, of *MITF* was detected in WS02‐III:1 which was predicted to be deleterious by MutationTaster. The same mutation was detected in the proband's mother, uncle (WS02‐II:2) and grandmother. Three of them only had brown freckles on the face but their hearing was normal. Another uncle of the proband (WS02‐II:3) with anormal phenotype did not have this mutation. A heterozygous deletion mutation, c.355_356insTCAGGCAGCGC, of *SOX10* was detected in WS03‐I:1 which was predicted to be deleterious by in silico analysis using MutationTaster, and other patients (WS03‐II:1 and II:2) also had the heterozygous mutation. Another son of the proband (WS03‐II:3) with normal phenotype did not have this mutation (Table 3; Figure 3).

Heterozygous mutations, c.1106_1107insTGGGGCCCCCCACACTA, c.511T>C (p.Y171H) and c.91_100 del in *SOX10* were detected in WS04‐II:2, WS05‐II:1, and WS06‐II:1, respectively, by NGS and confirmed by Sanger sequencing. c.1106_1107insTGGGGCCCCCCACACTA and c.91_100del were predicted to be deleterious by MutationTaster, c.511T>C (p.Y171H) was predicted to be deleterious by SIFT, PolyPhen‐2 and MutationTaster. The three proband's parents did not carry corresponding mutations as determined by Sanger sequencing indicating that the mutations occurred de novo (Table 3; Figure 3).

All of the six mutations have not been reported by previous studies or recorded in the public database (dbSNP, gnomAD, ESP, Clinvar, 1000 Genomes and ExAC) confirming the six mutations were novel. Moreover, these mutations were not found in unaffected family members or in 200 unrelated healthy control subjects.

**Figure 4:**
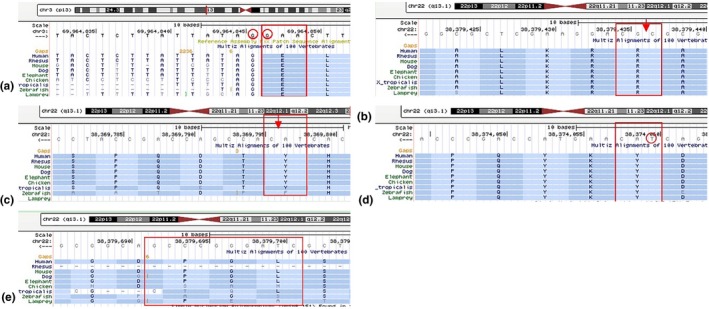
Conservation of amino acid sequences in the corresponding mutation of *MITF* and *SOX10* between species. The red rectangle represents the amino acid at the mutated site. The red circle represents the position of the mutant base. The red arrow indicates where the base is inserted. (a) c.859G>T (p.E287X) and c.859‐1G>A mutation in *MITF*. (b) c.355_356insTCAGGCAGCGC mutation in *SOX10*. (c) c.1106_1107ins TGGGGCCCCCCACACTA mutation in *SOX10*. (d) c.511T>C (p.Y171H) mutation in *SOX10*. (e) c.91_100del mutation in *SOX10*

Compared with homologous proteins of the mouse, rat, bovine, pig, dog, horse, and zebrafish, these six novel missense mutations identified in our study are conserved in other species (Figure 4).

## 4 DISCUSSION

Waardenburg syndrome, coined by Dutch ophthalmologist Petrus Johannes Waardenburg, is a neurocristopathy composed of hearing impairment and pigmentary abnormalities of eyes, skin and hair (Waardenburg, 1951). Its prevalence is estimated to be 1/42,000, and it is responsible for 1%–3% of total congenital deafness (Read & Newton, 1997). It is often described as an autosomal dominantly inherited disorder of neural crest cells (NCCs), but we now know that WS is clinically and genetically heterogeneous, and that not all forms are dominantly inherited.

Four subtypes of WS have been described thus far. WS type I (WS1; OMIM 193500) includes dystopia canthorum (an outward displacement of the inner canthi), and this feature distinguishes WS1 from WS type II (WS2; OMIM numbers 193510, 600193, 606662, 608890 and 611584 for 2A to 2E) (Pardono et al., 2003). WS type III (WS3; OMIM 148820) is similar to WS1 but includes musculoskeletal anomalies of the upper limbs. WS type IV (WS4; OMIM 277580, 613265, and 613266 for 4A to 4C) is similar to type I but has features of Hirschsprung disease (Wildhardt et al., 2013). Despite many efforts to clinically differentiate between the subtypes of WS by diagnostic criteria (Farrer et al., 1992), the rarity and highly varied expression has limited the ability to make an accurate diagnosis in individual patients. In addition, hearing loss and early graying are relatively common in the general population and are not specific to WS (Ouyang et al., 2009). Thus, the accuracy of WS diagnosis needs to be improved by using additional diagnostic procedures.

*MITF* is expressed in melanocytes and encodes a transcription factor containing the helix–loop−helix–leucine zipper structure. It is also a key factor in regulating the growth of melanocytes. Defects in melanocytes result in abnormal pigment distribution, whereas defects in melanocytes of the stria vascularis lead to WS2A (Curran, Raible, & Lister, 2009). *MITF* mutations are also observed in Tietz syndrome (albinism‐deafness syndrome) (Smith, Kelley, Kenyon, & Hoover, 2000). Most WS‐associated *MITF* mutations are located in exons 7–9 that correspond to the basic helix–loop−helix–Zip motifs (​Read & Newton, 1997; Tassabehji, Newton, & Read, 1994). The mutants may impair transcriptional activities, phosphorylation, the capacity of DNA binding and nuclear localization (Smith et al., 2000; Takeda et al., 2000; Wilkie, 1994). It seems more likely to result in haploinsufficiency of *MITF* function, with consequent downregulation of tyrosinase expression (Morell et al., 1997). *SOX10*, the *SRY*‐related transcription factor, binds to the *MITF* promoter and directly activates the expression of *MITF* (Verastegui, Bille, Ortonne, & Ballotti, 2000). Mutations in these two genes, therefore, are likely involved in the pathogenesis of WS2 through the same pathway and produce similar clinical phenotypes.

In this study, we identified two novel *MITF* heterozygous variations: c.859G>T (p.E287X) and c.859‐1G>A. The c.859G>T (p.E287X) mutation is a nonsense mutation in which the glutamic acid is replaced with a stop codon substitution at codon 287. The c.859‐1G>A is a splicing mutation. The two mutations are located in exon 9 of *MITF* at the intervening sequence of the leucine zipper and the third transactivation domain (AD3) of the protein. The two mutations were predicted to be disease‐causing by in silico analysis using Mutation Taster, with a *p*‐value of 1.0, suggesting a high possibility of protein functional alteration, which has not been previously reported. These variants co‐segregated with all affected individuals (WS01‐I:2, WS01‐II:3, WS02‐I:1, WS02‐II:1 and WS02‐II:2) of their families and were not observed in unaffected members (WS01‐II:1 and WS02‐II:3). Meanwhile, we identified four novel *SOX10* heterozygous variations: c.355_356insTCAGGCAGCGC, c.1106_1107insTGGGGCCCCCCACACTA, c.511T>C (p.Y171H) and c.91_100del. Three were frameshift mutations and one was a missense mutation. These mutations were predicted to be disease causing by in silico analysis using Mutation Taster. The c.355_356insTCAGGCAGCGC mutation detected in WS03‐I:2 co‐segregated with all affected individuals (WS03‐II:1 and WS03‐II:2) of the family and was not observed in unaffected members (WS03‐II:3). None of the proband's parents (WS04‐WS06) carried the corresponding mutations.

We subsequently verified that these six mutations did not exist in any of the widely used genomic databases (dbSNP, the 1000 Genomes Project, ExAC, gnomAD) and 200 unrelated healthy control subjects. According to the American College of Medical Genetics and Genomics (ACMG) standard and guidelines 2015 (Richards et al., 2015), the five variants (except *SOX10* c.511T>C) are pathogenic and their pathogenicity evidence grade PVS1 are high, and c.511T>C (p.Y171H) of *SOX10* is likely pathogenic (PS2+PM2+PP3).

In our study, three of the four WS2 probands (WS04‐06) with *SOX10* mutations were not detected in either of the proband's parents, suggesting that the mutations occurred de novo. By parental genotyping, we revealed an interesting inheritance pattern, as de novo mutations were frequently found in probands with *SOX10* mutations (3/4) but not in those with *MITF* mutations (0/2). The high de novo rates of *SOX10* mutations in WS2 may need special attention during the course of genetic diagnosis and counseling, as it can be initially mistaken as recessive inheritance prior to testing or be interpreted with over‐estimated recurrent risk without further parental testing.

In conclusion, we investigated the clinical and genetic characteristics of six families of WS2 in China. We identified six pathogenic variants in the *MITF* and *SOX10*, which have not been previously reported in the Chinese population. This report will contribute to a better understanding of the WS mutation spectrum identified so far. We also demonstrated that NGS is a useful tool for the identification of pathogenic gene variants in WS patients and for differentiation between WS and similar disorders. In addition to family genotyping, further functional studies are required to explore the genetic mechanism of these novel mutations.

## CONFLICT OF INTEREST

The authors declare no conflict of interest.
