# Supplementary material for: Identification of six novel variants in Waardenburg syndrome type II by next‐generation sequencing
Source: Mol Genet Genomic Med. 2020 Jan 20;8(3):e1128. doi: 10.1002/mgg3.1128 (PMC7057110; doi:10.1002/mgg3.1128)
Supplement: Supplementary file 1 [file MGG3-8-e1128-s001.docx]

Supporting information:

Table S1. Gene list of the NGS penal

| ACTG1 | ADGRV1 | ALX3 | BSND | CABP2 | CCDC50 | CDH23 |
| --- | --- | --- | --- | --- | --- | --- |
| CEACAM16 | CHD7 | CIB2 | CLDN14 | CLPP | CLRN1 | COCH |
| COL11A1 | COL11A2 | COL1A1 | COL1A2 | COL2A1 | COL4A3 | COL4A4 |
| COL4A5 | COL4A6 | COL9A1 | COL9A2 | CRYM | DFNA5 | DFNB59 |
| DIABLO | DIAPH1 | DIAPH3 | DSPP | ECM1 | EDN3 | EDNRB |
| ELMOD3 | ESPN | ESRRB | EYA1 | EYA4 | FGF3 | FGF8 |
| FGFR1 | FGFR3 | FLNA | FOXI1 | FREM1 | FXN | GATA3 |
| GIPC3 | GJB1 | GJB2 | GJB3 | GJB6 | GLYAT | GPSM2 |
| GRHL2 | GRXCR1 | HARS | HARS2 | HGF | HMX1 | HOXA2 |
| HSD17B4 | IL13 | ILDR1 | KARS | KCNE1 | KCNJ10 | KCNQ1 |
| KCNQ4 | KITLG | KRT9 | LAMA3 | LARS2 | LHFPL5 | LOXHD1 |
| LRTOMT | MARVELD2 | MIR96 | MITF | MPZ | MSRB3 | MYH14 |
| MYH9 | MYO15A | MYO1A | MYO1E | MYO3A | MYO6 | MYO7A |
| NDP | NDRG1 | NEFL | NELL2 | NF2 | OPA1 | OTOA |
| OTOF | OTOG | OTOGL | P2RX2 | PABPN1 | PAX3 | PCDH15 |
| PCDH9 | PDZD7 | PMP22 | PNPT1 | POLR1C | POLR1D | POU3F4 |
| POU4F3 | PROK2 | PROKR2 | PRPS1 | PTPN11 | PTPRQ | PTPRR |
| RDX | RPGR | SALL1 | SALL4 | SEC23A | SEMA3E | SERPINB6 |
| SIX1 | SIX5 | SLC17A8 | SLC19A2 | SLC26A4 | SLC26A5 | SMAD4 |
| SMPX | SNAI2 | SOX10 | STRC | TBC1D24 | TCIRG1 | TCOF1 |
| TECTA | TIMM8A | TJP2 | TMC1 | TMEM126A | TMIE | TMPRSS3 |
| TMPRSS4 | TNC | TPRN | TRIOBP | TRMU | TSPEAR | TYR |
| USH1C | USH1G | USH2A | WFS1 | WHRN | - | - |
